# Effect of Prolonged Coldness on Survival and Fertility of *Drosophila melanogaster*

**DOI:** 10.1371/journal.pone.0092228

**Published:** 2014-03-14

**Authors:** Robin J. Mockett, Yuri Matsumoto

**Affiliations:** Department of Biomedical Sciences, University of South Alabama, Mobile, Alabama, United States of America; CINVESTAV-IPN, Mexico

## Abstract

The laboratory fruit fly, *Drosophila melanogaster*, is used widely in biological research, but the requirement to maintain stocks with a roughly biweekly generation time imposes substantial burdens of labor, potential cross-contamination and mutation accumulation. The purpose of this study was to assess the impact of prolonged cold stress or milder cooling on survivorship and fertility. The hypothesis was that cold storage would result in postponement of reproduction and a longer generation time. Flies of several genotypes were maintained continuously at 4–11°C; recovery rates and subsequent yields of adult progeny were recorded. Adults and pupae of a relatively long-lived *y w* lineage were more resistant to severe cold stress than embryos and larvae. Adults exhibited minimal mortality up to at least 5 d at 4°C, 20 d at 8°C and 12 weeks at 11°C. Reproduction did not occur at these temperatures, but progeny were obtained after recovery at 25°C. At all temperatures, chilling caused a rapid, severe and progressive decrease in fertility during the first 2 d of recovery. The impact on fertility during the subsequent 2–4 d was much milder and it occurred only after prolonged incubation at low temperatures. The total reproductive output during the first 6 d of recovery was sufficient to replace the parental population after 12 weeks at 11°C. Food spoilage had an unexpectedly low impact on survivorship and fertility, and the reproductive output of F1 progeny was not affected by storing parental flies at 11°C for 8–10 weeks. In the case of *w* ^1118^ flies, replacement of the parents within 6 d of recovery was possible for up to 60 d at 11°C. Among less fertile genotypes, replacement of the parents was possible within 18 d after 4–10 weeks at 11°C. These results show that the 2-week maintenance interval of stocks of *D. melanogaster* can be extended 3–7 fold, at least for 1 generation, by storing adult flies at 11°C.

## Introduction

The fruit fly, *Drosophila melanogaster*, is among the most widely used model organisms in studies of biological phenomena ranging from genetics through development, physiology and behavior [1], [2]. The requirement to maintain stocks at room temperature on a roughly biweekly generation cycle is an impediment to research in all of these areas [3]. It necessitates continual stock maintenance, which is not only burdensome but also introduces a risk of cross-contamination among stocks. Furthermore, the mutation rate per generation is relatively high [4], [5], so both genotypes and phenotypes are subject to unwanted evolution as a function of time in the laboratory environment.

Stocks of many model organisms, notably the mouse and *Caenorhabditis elegans*, may be stored frozen at ultralow temperatures for extended intervals [3], [6]. In *Drosophila*, cryopreservation of stocks has not so far proven to be practical. An interval of roughly 3–4 h occurs beginning at 12–13 h of development, during which an appreciable fraction of embryos may survive cryopreservation [5], [7], [8]. Nevertheless, survival depends on the precise timing of exposure to 1-butanol in heptane for permeabilization, introduction of nontoxic cryoprotectant solutes up to ≥50% to induce vitrification, extremely rapid cooling (in a slush formed by applying a vacuum to liquid nitrogen) to avoid chilling injury, and rewarming at 100 000°C/min to prevent damage during devitrification [8]. These requirements are sufficiently demanding that cryopreservation has not become a routine method for long-term storage of fly stocks.

A potential alternative strategy to prolong the generation time and diminish the burden of stock maintenance arises from consideration of flies' overwintering strategies. Poikilothermic organisms in general have at least some capacity to resist adverse conditions, including low temperature, by entering a dormant state in which metabolic activity is minimized and reproduction is postponed [9]. In the case of *Drosophila*, chill tolerance varies dramatically among species [10] and strains [11], [12], with the commonly used laboratory *D. melanogaster* being relatively sensitive to cooling [10]. Nonetheless, adult *D. melanogaster* does exhibit a shallow reproductive diapause [12]. This phenomenon gives rise to the hypothesis that fly stocks could be stored for prolonged periods at temperatures below the threshold for reproduction, then rewarmed and allowed to reproduce.

Flies from a natural population in a cool, temperate environment have been shown previously to retain appreciable reproductive capability after storage for up to 5 months at 6°C [13], [14]. The question arises whether stocks that have been long acclimated to a laboratory environment would exhibit similar resistance to coldness. Reproductive diapause arrested senescence in flies recently obtained from wild populations but not in laboratory stocks, based on decreased survival times of the latter but not the former after returning from 11°C to 25°C [15]. The fecundity (egg-laying) of a stock maintained ∼13 years in the laboratory also declined but was not abolished after 6–9 weeks at 11°C [16]; however, the fertility of the eggs was not determined. Given that the proportion of fertile eggs diminishes as a function of adult age across the spectrum of temperatures (12–32°C) at which *D. melanogaster* reproduces [17], it remains to be determined whether viable populations could be recovered after holding laboratory stocks in diapause for prolonged time intervals.

The objectives of the study reported herein were to establish which developmental stages of a laboratory stock were most hardy at a low temperature, and then to quantify the ability of several stocks that vary widely in fertility under standard conditions to survive and generate adult progeny after recovering from progressively longer intervals of incubation at cool temperatures. The results show that the interval between stock maintenance efforts may be extended to approximately 6–14 weeks, albeit at the cost of diminished fertility upon return of the stocks to a permissive temperature.

## Materials and Methods

### 1. Fly Strains

The *y w*, *w* ^1118^ and 2S12i7Y *w* stocks used in this study have been described previously [18]. TM3, *ry* ^RK^ *Sb* ^1^ *Ser* ^1^/TM6B, *Tb* ^1^ flies were obtained from the Bloomington *Drosophila* Stock Center (Indiana University).

### 2. Medium

The medium used for all experiments contained 1.95% yeast (w/v), 1.63% sucrose, 6.93% cornmeal, 0.655% agar, 0.25% propionic acid (v/v) and 0.025% phosphoric acid. Unless noted otherwise, the medium contained 1.875 g/L methyl-4-hydroxybenzoate (methylparaben, MP) dissolved in 15 mL ethanol/L as a mold inhibitor.

### 3. Fly Collections and Incubation Conditions

Stocks were maintained and experimental flies developed at 25°C, ∼50% humidity and ambient lighting conditions. All collections of virgin, adult flies were performed under brief CO_2_ anaesthesia. Flies were housed in continuous darkness to minimize their activity [19] and at 80±8% humidity throughout all experiments at 4–11°C.

For comparisons of resistance to cold stress among developmental stages, embryos were collected daily on apple juice/agar plates and allowed to hatch. The first instar larvae (which were less susceptible to mechanical damage than embryos) were rinsed and transferred manually to food vials (10/vial). This procedure was repeated for 12 consecutive days, and additional vials of ≥10 embryos deposited directly onto the food were obtained on the final day. All developmental stages, ranging from freshly deposited embryos to adults (mixed sexes) up to 2 d posteclosion, were then placed at 4°C for 0–4 or 0–7 d (n = 4 vials per day of development per day of exposure to cold stress). The flies were then returned to 25°C and the number of adults that eclosed fully within a total of 14 d (before and after cold stress) at 25°C and recovered from cold-induced chill-coma was recorded.

For studies of adult survivorship at low temperatures and fertility following recovery at 25°C, flies were placed at 4–11°C beginning 0.5–3 d after eclosion. At 4°C or 8°C, groups of 3 *y w* virgin females and 3 males were housed separately in polystyrene vials containing the regular food medium. At 8°C, additional groups of unmated *y w* flies were housed in empty polystyrene vials or in polypropylene vials containing food. At 11°C, all experiments were performed in polystyrene vials containing food.

At 11°C (*y w*, Experiment 1, Results subsection 4, below), virgin females and males (3/vial) were either kept separately or crossed, placed immediately at 11°C, and left without further maintenance for intervals ranging from 0–12 weeks. A subset of flies was provided with fresh vials after 6 weeks and returned immediately to 11°C. Additional groups of unmated flies were held at 25°C and provided with fresh vials periodically. At weekly intervals, both crossed and unmated flies were returned from 11°C to 25°C, unmated flies from 11°C and 25°C groups were crossed, and all groups were transferred to fresh vials for tests of fertility.

All other experiments were performed with males and females housed together at 11°C, 3 per sex (*y w* and *w* ^1118^) or 5 per sex for stocks known to be less fertile (2S12i7Y *w* and TM3, *ry* ^RK^ *Sb* ^1^ *Ser* ^1^/TM6B, *Tb* ^1^). For *y w* (Experiment 2, Results subsection 5, below), flies were provided with 10 different diets containing varying amounts of the mold inhibitor (MP) and ethanol (Table 1) for 0 or 8 weeks, after which fertility was measured as described in section 2.4. *w* ^1118^ flies were placed immediately at 11°C for 0–84 d, and parallel groups were kept unmated at 25°C (10–40 d). At 9–14 d intervals, flies were returned from 11°C to 25°C, unmated flies at 25°C were crossed, and all groups were transferred to fresh vials. For 2S12i7Y *w* and TM3, *ry* ^RK^ *Sb* ^1^ *Ser* ^1^/TM6B, *Tb* ^1^, flies were held at 11°C for 0–12 weeks and a subset was returned to 25°C every 2 weeks.

**Table 1:** Media for 11°C: *y w* Experiment 2.

Medium	Methyl–4-hydroxybenzoate*	Ethanol*
i	1×	1×
ii	1×	2×
iii	1×	5×
iv	1×	10×
v	2×	2×
vi	5×	5×
vii	10×	10×
viii	2×	1×
ix	5×	1×
x	10×	1×

*Am°unts are fold differences in comparison with the standard medium containing 1.875 g/L methyl-4-hydroxybenzoate (dissolved in 15 mL ethanol/L).

### 4. Fertility

Reproductive output was measured at 25°C. Flies were crossed in fresh vials (n = 4–6) for 2 (4°C experiment) or 3 successive broods (8–11°C experiments), beginning immediately after the incubation of young adults (0.5–3 d posteclosion) at 4–11°C for 0–12 weeks. The duration of each brood was 2 d for *y w* and *w* ^1118^ flies, and 6 d for 2S12i7Y *w* and TM3, *ry* ^RK^ *Sb* ^1^ *Ser* ^1^/TM6B, *Tb* ^1^ flies. The sole exception was *y w* flies at 11°C (Experiment 2), for which fertility was tested on each diet (Table 1) after 0 weeks at 11°C (3×2 d broods), and in separate sets of flies under each of the following conditions after 8 weeks: (I) 3×2 d broods on fresh, standard medium, (II) 1×6 d brood on fresh, standard medium, and (III) 1×6 d in the same vials in which they had been stored at 11°C. In all cases, numbers of fully eclosed, adult F1 progeny were recorded within 14 d after parental flies were removed. Dead parents were not replaced between broods.

The fertility of F1 progeny was also determined on standard medium (3/sex/vial, 3×2 d broods) for *y w* parents housed at 11°C for 8 or 10 weeks (Experiment 1) and for all combinations of diet and brooding conditions after 8 weeks at 11°C (Experiment 2). In Experiment 1, parallel crosses were performed among F1 progeny of the flies that had been kept unmated for 8 weeks at 25°C, and among 1–2-day-old *y w* flies from stocks on a 2-week generation cycle.

### 5. Statistical analysis

Student's *t* tests, analyses of variance (ANOVA) and pairwise comparisons (Tukey tests) were performed using SYSTAT 12 software. The threshold of statistical significance was *P*<0.05.

## Results

### 1. Comparison of cold sensitivity among developmental stages: 4°C

**Figure 1:**
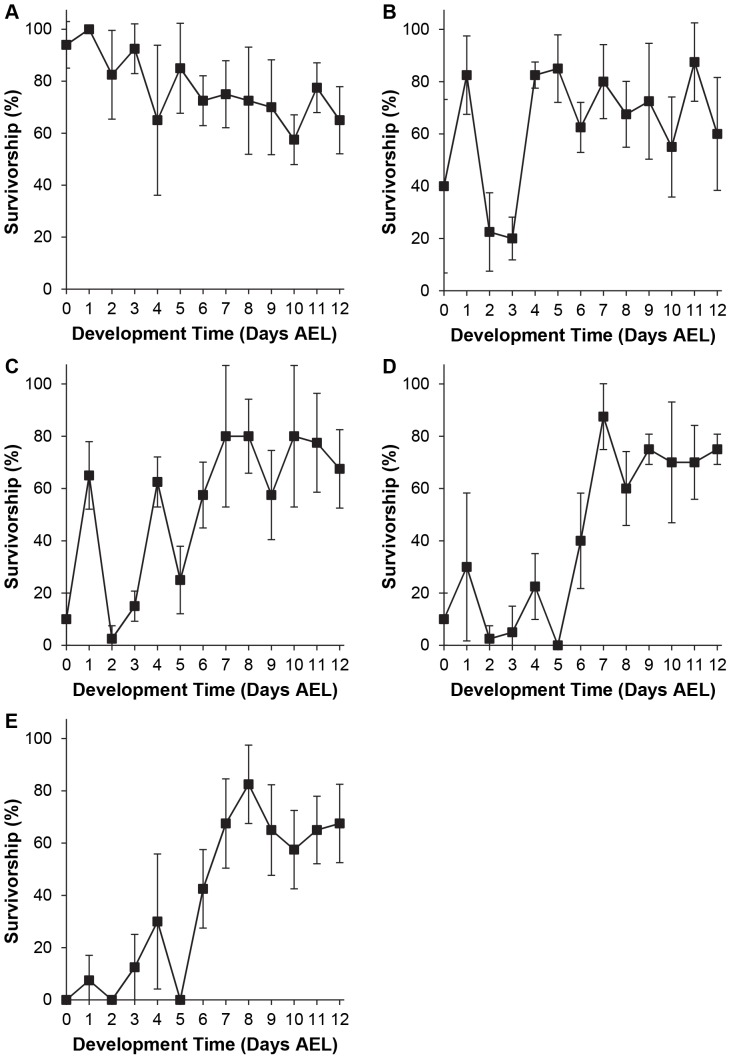
Survivorship of *y w* flies at different developmental stages after 0–4 d at 4°C. First instar larvae (10/vial) were collected from apple juice/agar plates up to 1 d after egg laying (AEL) and allowed to develop until 1–12 d AEL. Parental flies deposited ≥10 embryos (0 d AEL) directly onto the food surface for ∼2 h. The embryos and larvae were then placed simultaneously at 4°C for (A) 0 d, (B) 1 d, (C) 2 d, (D) 3 d or (E) 4 d. Results (mean ± S.D.; n = 4–5 vials for each development and exposure time) are percentages of fully eclosed adults after 14 d at 25°C, in addition to time spent at 4°C. For adults that eclosed prior to cold stress (none 0–9 d AEL and almost all flies 10–12 d AEL), the results are numbers that recovered after incubation at 4°C.

Initial experiments were performed to establish which developmental stages were most resistant to cooling, or whether all stages (embryos, larvae, prepupae, pupae and adults) could simply be housed together at low temperatures. Embryos and first instar larvae from a fertile, relatively long-lived *y w* lineage that is ancestral to most stocks in this laboratory were collected, reared at 25°C until 0–12 d after egg laying (AEL) and placed at 4°C for daily intervals ranging from 0–4 d. Survival to adulthood (or recovery of adult flies) was recorded after a total of 14 d at 25°C in addition to time spent at 4°C. Two-way ANOVA showed that both the duration of cold stress and developmental stage at the onset of cold stress affected survival (*P*<0.0005), and there was a strong interaction between these variables (*P*<0.0005). One-way ANOVA was next performed for each time interval at 4°C. Survivorship differed among developmental stages (0–12 d AEL) in each case (*P*<0.0005 for 1–4 d). Pairwise tests showed that survivorship differed minimally in the absence of cold stress (Fig. 1A), but it was significantly lower for groups 0, 2 and 3 d AEL relative to most other groups after 1–2 d at 4°C (Fig. 1B, C), and in nearly every comparison (70/72) of groups 0–5 d AEL *vs*. 7–12 d AEL after 3–4 d at 4°C (*P*<0.05; Fig. 1D, E). There was no difference in survivorship between any pair of pupal and adult groups (7–12 d AEL) placed at 4°C for 0–4 d.

This experiment was repeated 6 weeks later, except that flies were placed at 4°C for 0–7 d. The results were essentially the same, except that only 70% of pupae survived to adulthood and differences among developmental stages were only significant after 2–7 d at 4°C. Comparisons within each developmental stage showed that the survivorship of embryos (0 d AEL) was diminished after 1–2 d at 4°C, and no embryos placed at 4°C for longer than 2 d survived to adulthood. Larvae and prepupae (1–6 d AEL) were fairly resistant to placement at 4°C for 1 d, but survivorship was usually significantly diminished after 2–3 d and always after 4–7 d at 4°C. The blackened carcasses of dead larvae were prominent on the food surface, and visibly associated with deterioration of the food upon returning to 25°C. Pupae (7–9 d AEL) and adults that eclosed prior to cold stress (10–12 d AEL) did not have consistently decreased survivorship after 0–5 d at 4°C. Decreases after 6–7 d at 4°C (measured 8–12 d AEL) were not always significant. Thus, in general, resistance to 4°C cold stress was much greater for pupae and adults than for embryos, larvae and prepupae. Given that adult flies could be transferred more easily than pupae to fresh vials prior to cooling, adults were used to test the effect of longer-term cooling on survivorship and subsequent fertility.

### 2. Adult survivorship and fertility after incubation of y w flies at 4°C

**Figure 2:**
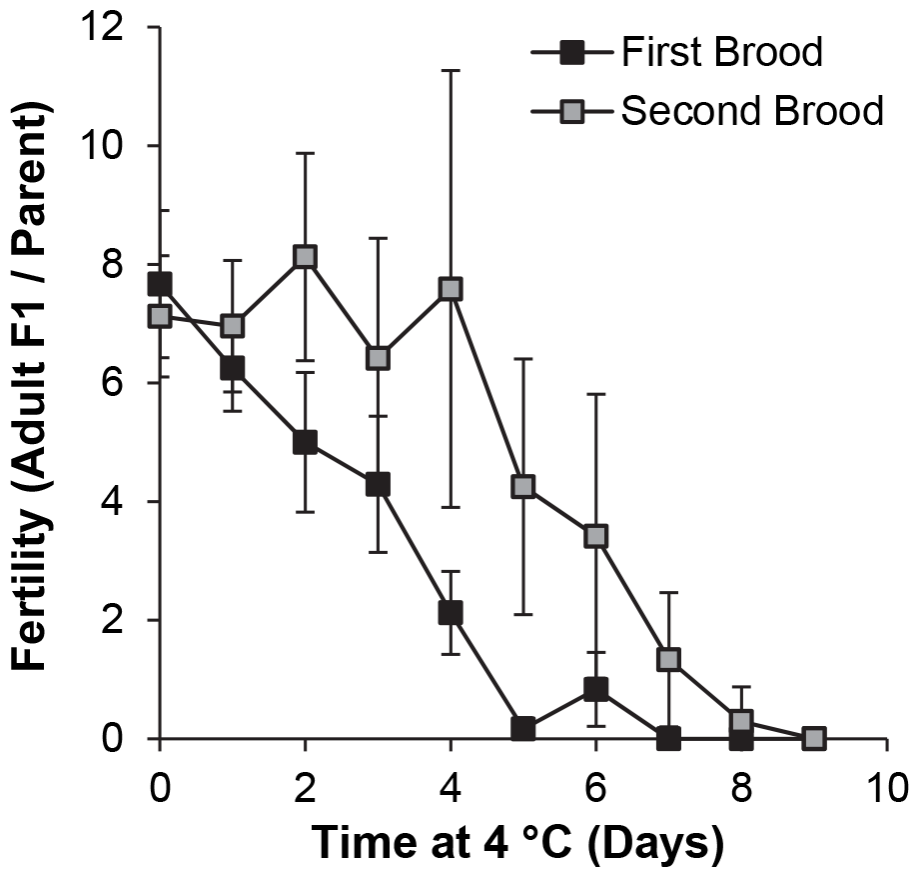
Fertility of adult *y w* flies after recovery from incubation at 4°C. Unmated flies were housed at 4°C for 0–9 d in groups of 3 females or 3 males, beginning 1 d after eclosion, then crossed at 25°C for two successive broods, each of 2 d duration. Results for both broods (mean ± S.D.) are numbers of adult F1 progeny per original parental fly (i.e. total progeny divided by 6). n = 4 vials per time point.

For studies of cold resistance and fertility in adult flies, the main question was whether and to what extent temporary exposure to coldness would impair fertility after the flies were returned to 25°C. Adult fertility was determined for *y w* flies following incubation at 4°C for 0–9 d. Fertility was determined over a 4 d interval to ensure that the flies had time to recover from the cold stress. This interval was subdivided into 2 broods, each of 2 d duration, to limit the number of F1 embryos and ensure that the number surviving to adulthood was not limited by crowding and exhaustion of food resources. The yield of fully eclosed, adult F1 progeny differed among times of incubation and between the first and second parental broods, and the interaction between these factors was significant (*P* = 0.001; Table 2). The fertility of the first brood decreased progressively to near zero between 0 and 5 d of cold stress (Fig. 2), and was significantly lower than at 0 d (control) after 2–9 d at 4°C (*P*≤0.001). In contrast, the fertility of the second brood was not affected at all until >4 d and was only significantly lower (*P*<0.005) after 7–9 d at 4°C. No progeny were obtained after 9 d and no parental flies recovered from 10–14 d continuous cold stress at 4°C. These results showed that 4°C cold stress was too severe for long-term storage of stocks and that cold storage did compromise fertility, but the magnitude of this effect diminished when the flies had >2 d to recover.

**Table 2:** Fertility (*y w*) After Recovery from 4°C Cold Stress.

Source of Variation	Type III SS*	df**	Mean Squares	F ratio	P value
Time of Exposure (0–9 d)	21 446.550	9	2 382.950	32.543	<0.0005
Brood (1–2)	2 645.000	1	2 645.000	36.122	<0.0005
Time×Brood	2 400.750	9	266.750	3.643	0.001
Error	4 393.500	60	73.225		

*Type III SS = adjusted sum of squares. **df = degrees of freedom.

### 3. Adult survivorship and fertility after incubation of y w flies at 8°C

The effect of a milder cold stress was tested next, on the assumption that it would allow flies to be stored longer before the impact on survivorship and subsequent fertility became prohibitive. *y w* flies were housed at 8°C for 0–20 d in polystyrene or polypropylene vials containing regular food medium, or in empty polystyrene vials to test the hypothesis that the absence of food would constitute a milder stress for inactive flies than food spoilage during prolonged cold storage. Polystyrene and polypropylene vials were compared to exclude the possibility that one type of plastic would impose a greater stress than the other, due for instance to the food drying more quickly and pulling away from the sides of the vials, or condensation on the sides of either type of vial forcing the adults to stand on the food surface. Given the results at 4°C, the number of broods was increased to 3 to extend the recovery interval. Mortality was negligible in all groups up to 10 d, after which it rose to 100% for males and >50% for females in empty vials by 18 d. In the presence of food, mortality at 20 d remained negligible in polystyrene vials and low (<20%) in polypropylene vials.

**Figure 3:**
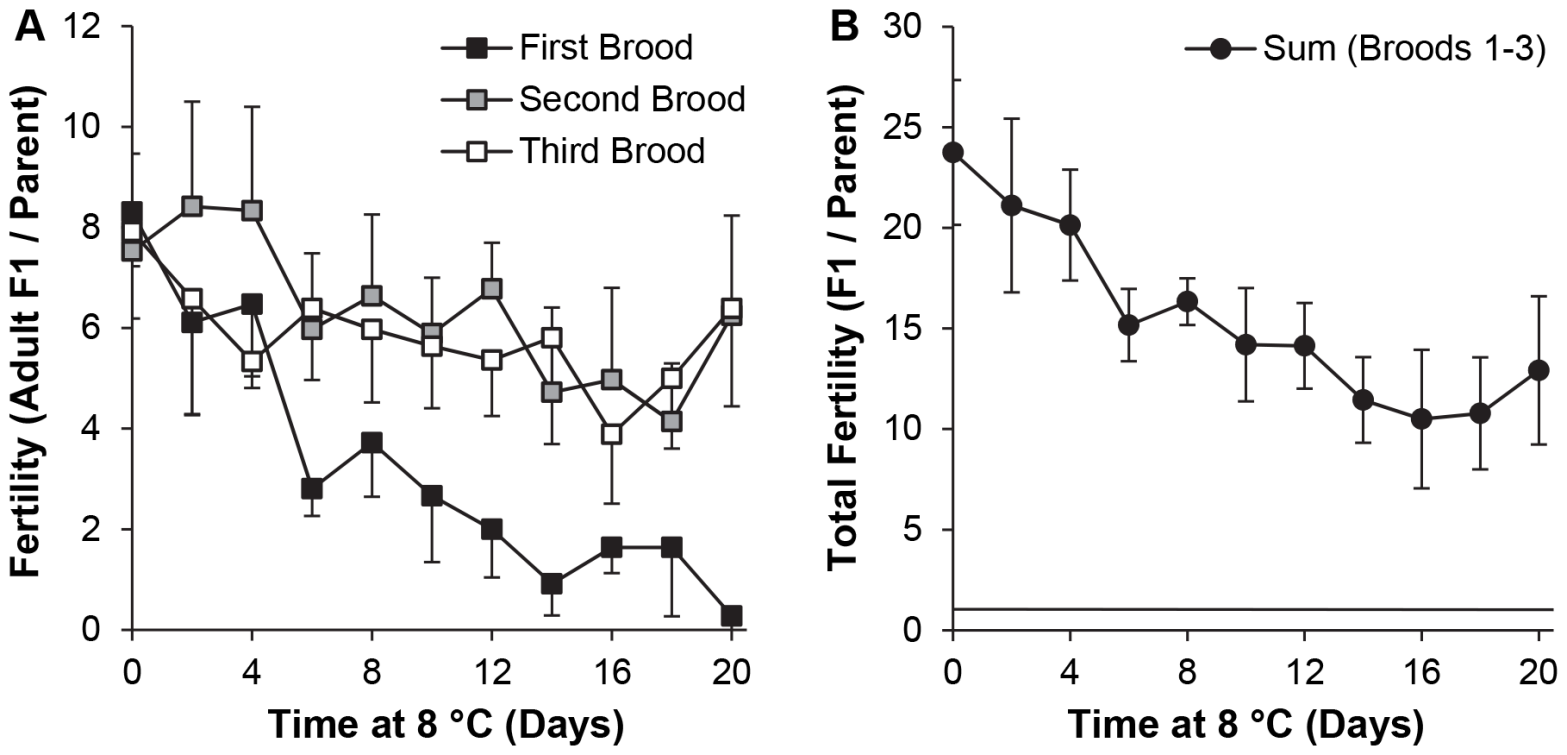
Fertility of adult *y w* flies after recovery from incubation at 8°C. Unmated flies were housed at 8°C for 0–20 d in groups of 3 females or 3 males, beginning 1 d after eclosion, then crossed at 25°C for 3 successive broods, each of 2 d duration. Results for all broods (mean ± S.D.) are numbers of adult F1 progeny per original parental fly (total progeny divided by 6). (A) Individual broods (B) The sum of progeny per parent from all 3 broods. The horizontal bar in (B) represents the threshold for stock replacement, i.e. 1.0 fully eclosed F1 adult per parental fly after 6 d. n = 6 vials per time point (3 polypropylene, 3 polystyrene).

Numbers of adult F1 progeny were counted and a 3-way ANOVA was performed, with time at 8°C (2–14 d), brood number (1–3) and housing condition at 8°C (polypropylene with food, polystyrene with or without food) as factors (Table 3). A separate 3-way ANOVA had time at 8°C (0–20 d), brood number and housing in polystyrene *vs*. polypropylene vials containing food as factors (Table 4). In both cases, time at 8°C, brood number and the interaction between these variables were highly significant (*P*<0.0005), but reproductive output did not differ significantly between flies kept with or without food (2–14 d; *P* = 0.6) or housed in different kinds of vials with food (0–20 d; *P* = 0.3), and there were no significant interactions between housing conditions and the other variables. Therefore, relationships among time at 8°C, brood number and reproductive output were determined using pooled data for flies with food in vials of both types. Fertility was found to be equal in each brood after 0–2 d at 8°C, lower in the third *vs*. second brood after 4 d, and significantly lower in the first than either the second or third brood after 6–20 d (Fig. 3A). The fertility of the first brood was strongly related to the duration of mild cold stress (*P*<0.0005). After 6–20 d at 8°C, the number of progeny was always lower than after 0–4 d (*P*<0.05 in 24/24 pairwise comparisons). In contrast, in the second and third broods, the relationship between fertility and duration of exposure to cold stress from 0–20 d was very weak: significant differences were observed in only 7/55 and 1/55 pairwise comparisons, respectively, and there was no difference among 0, 2 and 20 d groups. The cumulative reproductive output (broods 1–3) during the first 6 d after cold storage was >6 adult F1 progeny per parental fly for every vial, and >10 F1 per parent on average, regardless of incubation time at 8°C (Fig. 3B). The conclusions of this experiment were that: (i) the interval between generations of flies could indeed be increased at least 20 d by cold storage, (ii) the flies continued to need >2 d to recover fertility, even in response to milder cold stress, (iii) survivorship and subsequent fertility were not sensitive to the use of polystyrene *vs*. polypropylene vials, and (iv) food spoilage did not occur quickly enough to justify storing the flies without food.

**Table 3:** Fertility (*y w*) After Recovery from 8°C Cold Stress (2–14 d) With or Without Food.

Source of Variation	Type III SS*	df**	Mean Squares	F ratio	P value
Time at 8°C (2–14 d)	7 937.037	6	1 322.840	15.671	<0.0005
Brood (1–3)	9 358.487	2	4 679.243	55.433	<0.0005
Housing***	86.423	2	43.212	0.512	0.601
Time×Brood	4 165.439	12	347.120	4.112	<0.0005
Time×Housing	1 651.725	12	137.644	1.631	0.091
Brood×Housing	433.354	4	108.339	1.283	0.280
Time×Brood×Housing	1 556.942	24	64.873	0.769	0.769
Error	10 636.000	126	84.413		

*SS = sum of squares. **df = degrees of freedom.

***Housing was in polypropylene vials with food, or polystyrene vials with or without food.

**Table 4:** Fertility (*y w*) After Recovery from 8°C Cold Stress (0–20 d) With Food.

Source of Variation	Type III SS*	df**	Mean Squares	F ratio	P value
Time at 8°C (0–20 d)	14 101.162	10	1 410.116	16.529	<0.0005
Brood (1–3)	12 382.495	2	6 191.247	72.571	<0.0005
Housing***	92.045	1	92.045	1.079	0.301
Time×Brood	6 644.172	20	332.209	3.894	<0.0005
Time×Housing	608.010	10	60.801	0.713	0.711
Brood×Housing	97.727	2	48.864	0.573	0.565
Time×Brood×Housing	1 000.051	20	50.003	0.586	0.917
Error	11 261.333	132	85.313		

*SS = sum of squares. **df = degrees of freedom.

***Housing was in polypropylene or polystyrene vials with food.

### 4.1. Adult survivorship and fertility after incubation of y w flies at 11°C

Survivorship and subsequent fertility were next measured at 11°C, which was thought to be the mildest temperature cool enough to prevent egg-laying during the storage interval [17]. The length of storage compatible with subsequent recovery of a fertile stock was predicted to be longest at this temperature. To test the hypothesis that the flies would not reproduce at this temperature, and that the population density could be raised at least from 3 to 6 (3 pairs) per vial without accelerating food spoilage, a subset of females and males were crossed immediately before cooling and housed together at 11°C, while others were stored separately and crossed immediately after their return to 25°C.

Mortality at 11°C was negligible, i.e. 0.3% throughout the experiment. Specifically, only 1 female fly died after 5 weeks, and 1 male after 6 weeks. Small mold spots appeared on the food surface beginning at 6 weeks, at which time some flies were provided with fresh vials. Flies maintained at 25°C began to die after 7–8 weeks, so flies from multiple vials at 25°C were combined to give 3 females and 3 males at the beginning of the fertility test. Throughout the experiment, all vials containing females and males together at 11°C were retained at 25°C for another 12–14 d after the flies had been removed. No eggs were observed, and there was never any sign of development of progeny in any of these vials.

A 3-way ANOVA restricted to flies housed at 11°C in the same vial for 1–12 weeks demonstrated that (i) the length of incubation at 11°C, brood number and interaction between these two factors were all highly significant (*P*≤0.001), but (ii) crossing the flies before *vs*. after incubation at 11°C had no impact on their subsequent fertility and none of the interaction terms involving housing were significant (Table 5). A separate 3-way ANOVA showed that time at 11°C (8–12 weeks) and brood number had significant effects (*P*<0.0005), provision of new vials after 6 weeks at 11°C had no main effect on reproductive output at 8–12 weeks, but some significant interactions were observed (Table 6). The flies provided with fresh vials after 6 weeks were not included in the remaining analyses.

**Table 5:** Fertility (*y w*) After Housing Unmated or Crossed Flies at 11°C (1–12 Weeks).

Source of Variation	Type III SS*	df**	Mean Squares	F ratio	P value		
Time at 11°C (1–12 Weeks)	53 018.516	9	5 890.946	50.659	<0.0005		
Brood (1–3)	18 007.635	2	9 003.818	77.428	<0.0005		
Housing***	57.191	1	57.191	0.492	0.484		
Time×Brood	5 425.007	18	301.389	2.592	0.001		
Time×Housing	1 355.912	9	150.657	1.296	0.242		
Brood×Housing	357.510	2	178.755	1.537	0.218		
Time×Brood×Housing	795.549	18	44.197	0.380	0.990		
Error	19 885.083	171	116.287				

*SS = sum of squares. **df = degrees of freedom.

***Housing at 11°C: males and females in separate vials or together.

**Table 6:** Fertility (*y w*) With or Without Fresh Vials After 6 Weeks at 11°C (8–12 Weeks)†.

Source of Variation	Type III SS*	df**	Mean Squares	F ratio	P value		
Time at 11°C (8–12 Weeks)	5 475.097	2	2 737.548	30.575	<0.0005		
Brood (1–3)	7 196.704	2	3 598.352	40.190	<0.0005		
Vials (Fresh or not, 6 Weeks)	101.824	1	101.824	1.137	0.288		
Time×Brood	4 099.564	4	1 024.891	11.447	<0.0005		
Time×Vials	837.111	2	418.555	4.675	0.011		
Brood×Vials	704.097	2	352.049	3.932	0.022		
Time×Brood×Vials	1 255.397	4	313.849	3.505	0.010		
Error	10 475.525	117	89.534				

† : In error, Brood 3 in new vials (10 weeks) was extended to 4 d (*vs*. 2 d for all other broods). Data are shown for all progeny. Dividing the number of progeny in the extended brood by 2 caused the Brood×Vials interaction to lose significance (*P* = 0.101), but no other *P* values rose above or fell below the significance threshold as a result.

*SS = sum of squares. **df = degrees of freedom.

**Figure 4:**
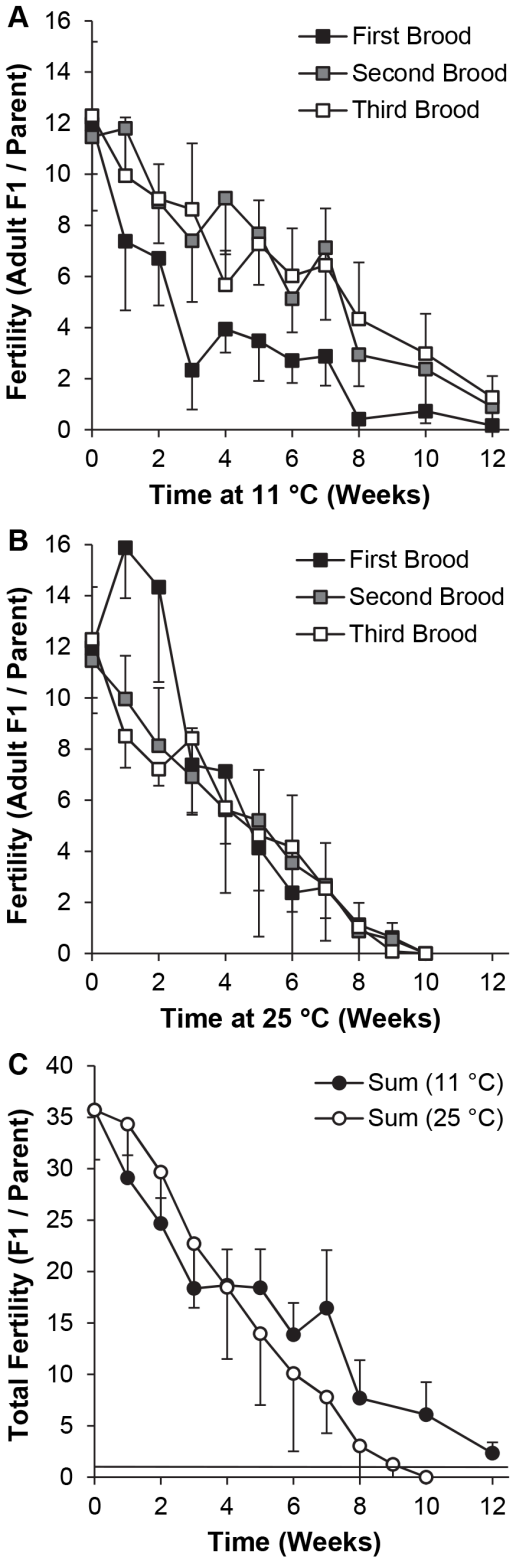
Fertility of adult *y w* flies after recovery from incubation at 11°C. Unmated flies were housed at 11°C for 0–12 weeks or 25°C for 0–10 weeks in groups of 3 females (beginning 1 d after eclosion) or 3 males (0.5 d after eclosion), then crossed at 25°C for 3 successive broods, each of 2 d duration. Alternatively, the flies were crossed immediately before housing at 11°C. Results for all broods (mean ± S.D.) are numbers of adult F1 progeny per original parental fly (total progeny divided by 6). (A) 11°C. n = 8 vials / treatment (4 crossed before and 4 crossed after incubation at 11°C). (B) 25°C. n = 4 vials / treatment. n = 12 vials at 0 weeks. (C) Sums of progeny per parent from all 3 broods at 11°C or 25°C. The horizontal bar in (C) represents the threshold for stock replacement, i.e. 1.0 fully eclosed F1 adult per parental fly after 6 d.

A 3-way ANOVA with temperature (11°C *vs*. 25°C), incubation time (1–10 weeks) and brood number as factors showed that all main effects and interactions were significant (Table 7). Comparisons among times at 11°C (Fig. 4A) showed that fertility was lower than at 0 weeks (*P*<0.05) after 1–12 weeks (first brood), 3, 5–12 weeks (second brood) and 2–12 weeks (third brood). After incubation at 25°C (Fig. 4B), fertility was lower than at 0 weeks only after 3–10 weeks (first and second broods) and 2, 4–10 weeks (third brood). Too few flies survived to measure fertility after 12 weeks at 25°C. Comparisons among broods after incubation at 11°C revealed that fertility was lower in the first than in both the second brood (1–8 weeks) and the third brood (2–3, 5–12 weeks). Fertility did not differ among broods after incubation at 25°C for 0 or 3–10 weeks, and it was higher in the first than both the second and third broods after 1–2 weeks (Figs. 4A, B). Comparisons between temperatures showed that fertility was lower after incubation at 11°C than 25°C for the first but not the second or third brood after 1–4 weeks. It was higher in the second brood after 4, 7 and 8 weeks, and in the third brood after 2, 5, 7 and 8 weeks. After 10 weeks at 11°C, the number of progeny was significantly greater than 0 for all broods, but no progeny were generated in any vial held at 25°C.

**Table 7:** Fertility (*y w*) After Housing Flies at 11°C *vs*. 25°C (1–10 Weeks).

Source of Variation	Type III SS*	df**	Mean Squares	F ratio	P value
Temperature (11°C *vs*. 25°C)	618.347	1	618.347	4.730	0.031
Time (1–10 Weeks)	90 885.920	8	11 360.740	86.896	<0.0005
Brood (1–3)	2 448.012	2	1 224.006	9.362	<0.0005
Temperature×Time	7 010.389	8	876.299	6.703	<0.0005
Temperature×Brood	13 273.444	2	6 636.722	50.763	<0.0005
Time×Brood	8 199.265	16	512.454	3.920	<0.0005
Temperature×Time×Brood	5 289.611	16	330.601	2.529	0.001
Error	35 299.625	270	130.739		

*SS = sum of squares. **df = degrees of freedom.

The cumulative reproductive output from eggs laid during the first 6 d after mating (the sum of all 3 broods) was 36±5 adult F1 progeny (mean ± S.D., n = 12 vials) for young parents at 25°C (0 weeks incubation at 11°C). It remained above the replacement level, i.e. ≥1 adult F1 fly per parent, for every vial from 1–8 weeks at 11°C, 15/16 vials after 10 weeks and 7/13 vials after 12 weeks (Fig. 4C). When flies were held as virgins at 25°C, cumulative fertility initially declined more slowly than at 11°C, but it only attained the replacement level for all vials after 1–7 weeks. The results showed that: (i) the interval between generations of the *y w* stock could be extended up to 12 weeks (and safely up to 8–10 weeks) by storage at 11°C, (ii) the length of the storage interval was inversely related to subsequent fertility, (iii) flies continued to need >2 d to recover their fertility after cooling, (iv) cooling for short intervals impaired subsequent fertility more severely than the normal aging of young virgin flies, but subsequent attrition of fertility due to cooling was milder than the effect of aging, and (v) separation of males and females prior to cooling was not necessary to prevent egg laying or decelerate any effect of food spoilage on reproduction.

### 4.2. Fertility of F1 progeny after incubation of y w parents at 11°C

Preservation of a viable stock depends not only on the generation of progeny, but also on their viability. Development of F1 embryos to adulthood was taken as a minimal standard of stock viability, but the fertility of the progeny was also tested. For F1 progeny of parents housed at 11°C for 8 weeks, the cumulative reproductive output from eggs laid during the first 6 d after mating was 38±6 F2 adults per F1 fly (n = 94 vials). For parents housed at 11°C for 10 weeks, the output was 33±5 F2 per F1 fly (n = 43 vials). These values were statistically indistinguishable from the yield of F1 progeny from parental flies after 0 weeks incubation. Housing the parental males and females together (or apart) and providing fresh vials at 6 weeks (or not) had no direct effect on the fertility of F1 flies at 8 weeks or 10 weeks, although there was a significant interaction between these variables at 8 weeks. After 8 weeks, F1 fertility was slightly higher when parental flies had been kept unmated in old vials at 11°C than when they had been kept together in old vials or when young parents were obtained from stock bottles, but no different than the fertility of F1 progeny of parents housed at 25°C for 8 weeks before mating. Therefore, notwithstanding the decreased number of F1 progeny due to prolonged cooling of the parents, the F1 flies that survived were unimpaired with respect to fertility.

### 5. Adult survivorship, fertility and F1 fertility after incubation of y w flies on different media at 11°C

Given the observation of mold spots after storage intervals >6 weeks, it was hypothesized that the food medium could be optimized to improve reproductive output after cold storage. Flies were incubated at 11°C for 0 or 8 weeks on ten different media containing 1–10×standard quantities of the mold inhibitor (MP) and ethanol (Table 1). Total mortality after 8 weeks ranged from 1% (medium i) to 8% (media vi and vii). At both 0 and 8 weeks, MP crystals were observed in media vi and vii, which contained 5–10× MP and 5–10× ethanol. All other media were slightly sticky and contained small mold spots after housing flies for 8 weeks, except that medium v (2× MP, 2× ethanol) contained no visible crystals and no mold spots.

At 0 weeks, the total reproductive output after 6 d (3×2 d broods) on regular food (medium i) was 25.3±2.2 adult F1 progeny per parent. One-way ANOVA revealed significant differences in numbers of progeny among media (*P*<0.0005). Pairwise comparisons showed that fertility was higher on media i, ii, v, viii and x than on media iv, vi and vii (*P*<0.05). In comparison with medium i, development of F1 progeny was delayed and cumulative parental fertility was diminished 42–63% on media iv, vi and vii. A 6 d brood on treatment i yielded only 9.9±0.8 F1 progeny per parent, i.e. 61% fewer than 3×2 d broods.

After 8 weeks, fertility was tested under three conditions simultaneously: (I) 3×2 d broods in new vials (medium i), (II) 1×6 d brood in new vials (medium i), and (III) 1×6 d in old vials used to store the flies at 11°C. Vials with dead flies were excluded or pooled (media vi and vii) to obtain identical numbers of parents for comparisons of fertility among all media for conditions I–III. The total reproductive output after 6 d on regular food (condition I, medium i) was 7.3±2.8 adult F1 progeny per parent, i.e. 71% lower than at 0 weeks. Based on 2-way ANOVA (Table 8), the total reproductive output was statistically indistinguishable among conditions I-III and condition×medium interaction was not significant, but there were significant differences among the media. Pairwise comparisons showed that the yield of progeny was lower on medium vii *vs*. ii, iii and ix, lower on medium vi *vs*. ix (*P*<0.05), and no other differences were significant. Within condition I (3×2 d broods), fertility differed substantially among media and broods, but there was no interaction between these variables (Table 9). With brood as a covariate, the yield of progeny was lower on medium vii *vs*. i, ii, iii, viii, ix and x (*P*<0.05). With medium as a covariate, the second and third broods yielded 2.9 and 3.9 fold more progeny than the first brood, respectively (*P*<0.0005). The total reproductive output after 6 d on standard medium surpassed the population replacement level of 1.0 progeny per parent in 4/4, 3/4 and 7/8 vials for conditions I, II and III, respectively.

**Table 8:** Fertility (*y w*) After 8 Weeks at 11°C on Different Media.

Source of Variation	Type III SS*	df**	Mean Squares	F ratio	P value
Condition (I–III)	1 680.200	2	840.100	2.356	0.101
Medium (i–x)	14 831.175	9	1 647.908	4.621	<0.0005
Condition×Medium	7 738.800	18	429.933	1.206	0.274
Error	32 095.750	90	356.619		

*SS = sum of squares. **df = degrees of freedom.

**Table 9:** Fertility (*y w*) After 8 Weeks at 11°C – Condition I.

Source of Variation	Type III SS*	df**	Mean Squares	F ratio	P value
Medium (i–x)	1 830.242	9	203.360	4.810	<0.0005
Brood (1–3)	2 680.467	2	1 340.233	31.699	<0.0005
Medium×Brood	461.033	18	25.613	0.606	0.886
Error	3 805.250	90	42.281		

*SS = sum of squares. **df = degrees of freedom.

F1 progeny were collected for all 10 treatments under conditions I–III and crossed 1–4 d after eclosion for 3×2 d broods. For condition I, medium i, the cumulative reproductive output from eggs laid during the first 6 d after mating was 37±8 F2 adults per F1 fly (n = 4 vials), i.e. the same as for parents (0 weeks) and F1 progeny (8 weeks) in Experiment 1 (subsection 4, above). The fertility of F1 flies differed significantly based on parental housing conditions, medium and brood number, and both condition×medium and condition×brood interactions were significant (Table 10). Conditions I–III yielded statistically indistinguishable numbers of F2 progeny in all broods on media i, v, ix and x, but condition I yielded more progeny than II or III in 1 or 2 broods from each of the other media. Comparisons among parental media within each condition, with brood number as a covariate, revealed no significant differences except for greater F1 fertility for parents on media i, ii and vi *vs*. iii (condition II) and iv *vs*. viii and x (condition III). Comparisons among broods within each condition, with medium as a covariate, showed that the first brood of F1 flies always yielded more F2 progeny than the second and third broods (*P*<0.0005), and the second brood yielded more than the third brood (*P*≤0.001).

**Table 10:** F1 Fertility (*y w*) After Housing Parents at 11°C for 8 Weeks on Different Media.

Source of Variation	Type III SS*	df**	Mean Squares	F ratio	P value
Condition (I–III)	8 813.185	2	4 406.593	30.876	<0.0005
Medium (i–x)	3 053.882	9	339.320	2.378	0.013
Brood (1–3)	60 188.401	2	30 094.201	210.863	<0.0005
Condition×Medium	5 216.493	18	289.805	2.031	0.009
Condition×Brood	4 098.411	4	1 024.603	7.179	<0.0005
Medium×Brood	2 500.533	18	138.919	0.973	0.491
Condition×Medium×Brood	3 946.702	36	109.631	0.768	0.829
Error	37 535.083	263	142.719		

*SS = sum of squares. **df = degrees of freedom.

Collectively, the results of this experiment confirmed that prolonged cooling decreased subsequent reproductive output, and that its effect was most pronounced during the first 2 d after the flies' return to 25°C. Once again, the surviving F1 flies were not compromised in their fertility (or size or vigor, based on visual inspection). Subdividing the 6 d recovery interval into 3 broods was useful for the purposes of this experiment, but not necessary to maximize the number of F1 flies during future stock maintenance. The standard amount of mold inhibitor was sufficient to preserve the food well enough that even the provision of a fresh vial after storage was not essential.

### 6. Adult survivorship and fertility after incubation of w^1118^ flies at 11°C

The hypothesis that prolonged cooling could be used to prolong the generation time was next tested in *w* ^1118^ flies, which are comparable in longevity and fertility to standard strains such as Oregon R and Canton S [18]. Results for the *w* ^1118^ strain followed the same trends as for *y w* flies. Mortality was <5% from 10–84 d at 11°C. The vials contained numerous mold spots by 60–70 d at 11°C, but the flies were not visibly affected. Again, after 14 d at 25°C, no progeny developed in any of the vials in which females and males had been stored together at 11°C.

**Figure 5:**
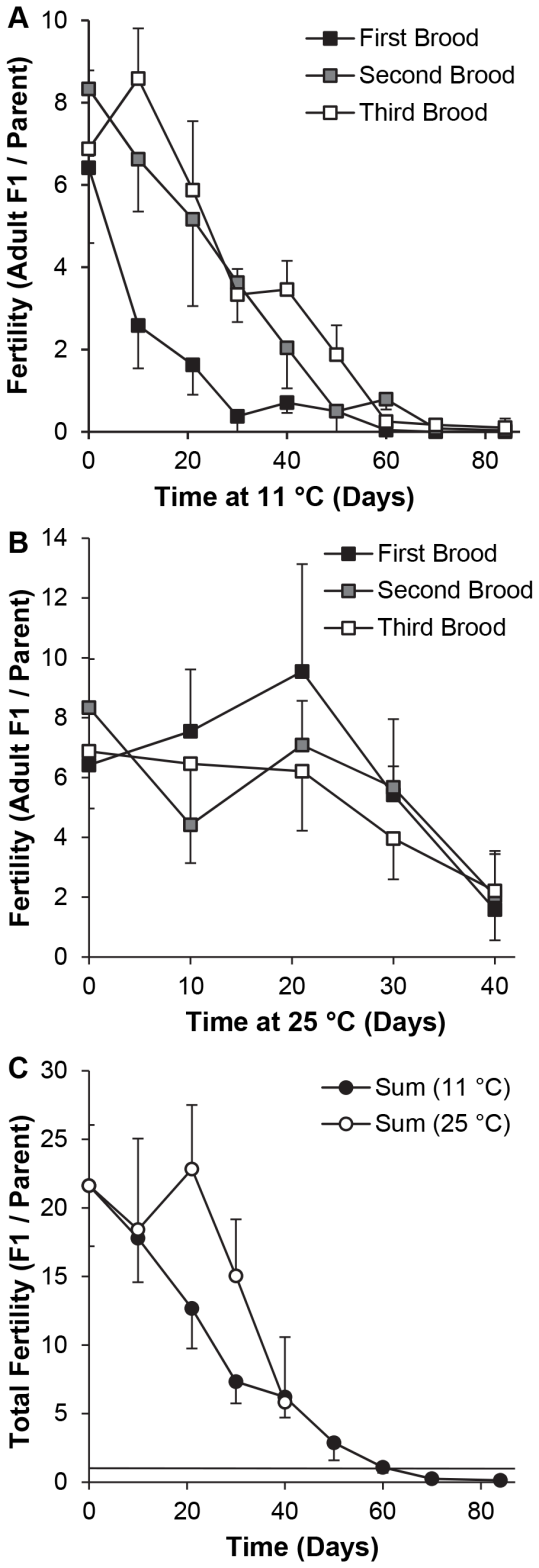
Fertility of adult *w* ^1118^ flies after recovery from incubation at 11°C. Within 1 d after eclosion, groups of 3 females and 3 males were crossed and placed immediately at 11°C for 0–84 d. Unmated flies were housed at 25°C for 10–40 d, and then crossed. All flies were then provided with fresh vials for 3 successive broods at 25°C, each of 2 d duration. Results for all broods (mean ± S.D.) are numbers of adult F1 progeny per original parental fly (total progeny divided by 6). n = 4 vials per time point, except n = 8 at 0 weeks and n = 5 at 12 weeks. (A) 11°C. (B) 25°C. (C) Sums of progeny per parent from all 3 broods at 11°C or 25°C. The horizontal bar in (C) represents the threshold for stock replacement, i.e. 1.0 fully eclosed F1 adult per parental fly after 6 d.

Cumulative reproductive output of young *w* ^1118^ parents (0 weeks incubation) during the first 6 d after mating was 22±4 adult F1 progeny (mean ± S.D., n = 8 vials). A 3-way ANOVA revealed significant differences in fertility between incubation temperatures (11°C *vs*. 25°C) from 10–40 d, among time points over this interval, and among broods, as well as significant temperature×time and temperature×brood interactions (Table 11). Fertility was lower after 11°C *vs*. 25°C incubation during the first brood (10–30 d) and second brood (30 d), but not the third brood. Comparisons among incubation times at 11°C (0–84 d) demonstrated significant effects of both time and brood number, and significant time×brood interaction (Fig. 5A; Table 12). The fertility of the first brood decreased exponentially, falling by 59% within the first 10 d at 11°C (*P*<0.0005), and was close to zero from 30–84 d. The fertility of the second brood fell 20% in the first 10 d, and continued to decrease linearly to a minimal level after 50 d. The fertility of the third brood did not decrease at all during the first 10 d, was significantly decreased only after 30 d, and reached a minimal level only after 60 d. At 25°C, fertility was significantly diminished after 30–40 d (Fig. 5B; Table 13), but there was no difference among broods and the time×brood interaction was not significant.

**Table 11:** Fertility (*w* ^1118^) After Housing Flies at 11°C *vs*. 25°C (10–40 d).

Source of Variation	Type III SS*	df**	Mean Squares	F ratio	P value
Temperature (11°C *vs*. 25°C)	1 971.094	1	1 971.094	18.442	<0.0005
Time (10–40 d)	9 631.365	3	3 210.455	30.038	<0.0005
Brood (1–3)	1 077.062	2	538.531	5.039	0.009
Temperature×Time	1 948.365	3	649.455	6.077	0.001
Temperature×Brood	4 545.437	2	2 272.719	21.265	<0.0005
Time×Brood	810.854	6	135.142	1.264	0.285
Temperature×Time×Brood	1 092.479	6	182.080	1.704	0.133
Error	7 695.250	72	106.878		

*SS = sum of squares. **df = degrees of freedom.

**Table 12:** Fertility (*w* ^1118^) After Housing Flies at 11°C (0–84 d).

Source of Variation	Type III SS*	df**	Mean Squares	F ratio	P value
Time (0–84 d)	33 363.654	8	4 170.457	96.293	<0.0005
Brood (1–3)	3 285.825	2	1 642.912	37.934	<0.0005
Time×Brood	3 473.425	16	217.089	5.012	<0.0005
Error	4 157.750	96	43.310		

*SS = sum of squares. **df = degrees of freedom.

**Table 13:** Fertility (*w* ^1118^) After Housing Flies at 25°C (0–40 d).

Source of Variation	Type III SS*	df**	Mean Squares	F ratio	P value
Time (0–40 d)	9 861.458	4	2 465.365	16.523	<0.0005
Brood (1–3)	374.111	2	187.056	1.254	0.293
Time×Brood	2 192.083	8	274.010	1.836	0.089
Error	8 504.750	57	149.206		

*SS = sum of squares. **df = degrees of freedom.

The cumulative reproductive output of the first three broods was above the replacement level of ≥1 adult F1 fly per parent for 4/4 vials at each time point from 10–50 d at 11°C, and on average after 60 d (Fig. 5C). After 60–84 d at 11°C, a fourth brood of 6 d duration was also made. At 60 d, the fourth broods each yielded more than enough progeny to replace the parents, and the cumulative reproductive output of the 4 broods ranged from 2.8 – 7.3 adult F1/parent (n = 4) over a 12 d interval. At 70–84 d, the fertility of the fourth brood was also minimal, and the cumulative reproductive output was only 0.0 – 1.2 F1/parent. This experiment showed that cooling could be used to prolong the generation time in more than one genotype, but the maximum time of storage compatible with recovery of the stock was slightly shorter for *w* ^1118^ than for *y w* flies.

### 7. Adult survivorship and fertility after incubation of 2S12i7Y w and TM3, ry^RK^ Sb^1^ Ser^1^/TM6B, Tb^1^ flies at 11°C

A final objective was to test genotypes that are normally among the least fertile in the laboratory. 2S12i7Y *w* has lifetime reproductive output only 6–11% as great as *w* ^1118^ at 25°C [18], and TM3, *ry* ^RK^ *Sb* ^1^ *Ser* ^1^/TM6B, *Tb* ^1^ requires frequent monitoring of population density to avoid stock extinction. When flies from these stocks were held at 11°C, mortality was <20%, except for TM3, *ry* ^RK^ *Sb* ^1^ *Ser* ^1^/TM6B, *Tb* ^1^ at 12 weeks (22%), and dead flies were not replaced prior to fertility experiments. Again, no progeny were generated at 11°C.

**Figure 6:**
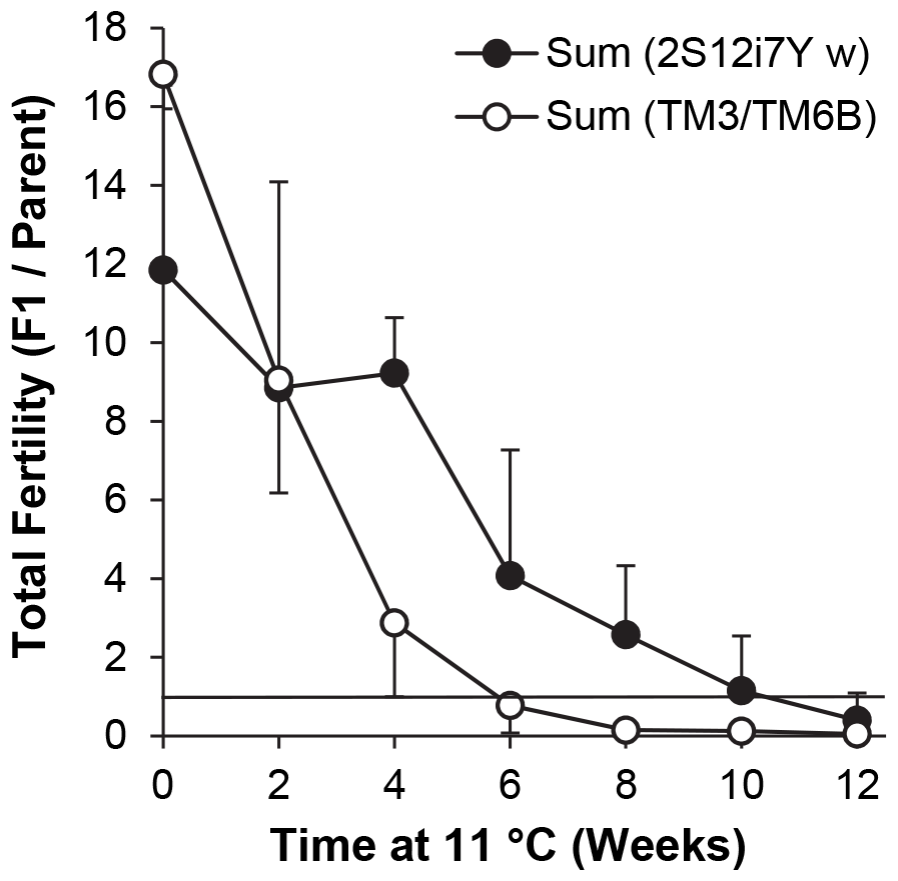
Fertility of adult flies from less fertile stocks after recovery from incubation at 11°C. Groups of 5 female and 5 male flies were crossed 1–3 d after eclosion and placed immediately at 11°C for 0–12 weeks. Flies were then provided with fresh vials for 3 successive broods at 25°C, each of 6 d duration. Results (mean ± S.D.) are sums of adult F1 progeny per original parental fly (total progeny divided by 10) from all 3 broods. n = 4 vials per time point (except for 2S12i7Y *w* at 12 weeks, n = 3). Filled circles: 2S12i7Y *w*. Open circles: TM3, *ry* ^RK^ *Sb* ^1^ *Ser* ^1^/TM6B, *Tb* ^1^. The horizontal bar represents the threshold for stock replacement, i.e. 1.0 fully eclosed F1 adult per parental fly after 18 d.

Like the other genotypes, 2S12i7Y *w* exhibited a significant time-dependent decline in total reproductive output (Fig. 6; Table 14); however, there were no significant differences among the 3 6-d broods, and no interaction between storage time and brood. There was no difference in total fertility from 0–4 weeks at 11°C, after which there was a decrease at 6 weeks (*P*<0.05 for all pairwise comparisons, 0–4 *vs*. 6–12 weeks). Reproductive output continued to trail off from 6–12 weeks, but the decline in residual fertility was not statistically significant. The 10 parental flies were able to generate 10 adult progeny within 18 d at 25°C in all 4 replicate vials after 0–4 weeks at 11°C, 3/4 vials after 6–8 weeks, 2/4 vials after 10 weeks and 1/3 vials after 12 weeks.

**Table 14:** Fertility (2S12i7Y *w*) After Housing Flies at 11°C (0–12 Weeks).

Source of Variation	Type III SS*	df**	Mean Squares	F ratio	P value
Time (0–12 Weeks)	15 799.455	6	2 633.242	17.684	<0.0005
Brood (1–3)	600.591	2	300.295	2.017	0.142
Time×Brood	2 502.109	12	208.509	1.400	0.192
Error	8 785.250	59	148.903		

*SS = sum of squares. **df = degrees of freedom.

The fertility of TM3, *ry* ^RK^ *Sb* ^1^ *Ser* ^1^/TM6B, *Tb* ^1^ flies decreased nearly exponentially at 11°C (Fig. 6). The time of incubation at 11°C, brood number and time×brood interaction were all significant (*P*<0.02; Table 15). The first 6 d brood was more fertile than the others only at 0 weeks (*P*<0.02), and the reproductive output of every brood diminished from 0–2 weeks (*P*<0.01) and from 2–4 weeks (*P*<0.03). Although a few flies were able to produce adult progeny even after 12 weeks, the reproductive output of the population as a whole fell below the replacement level after 6 weeks. The decrease in fertility (number of progeny) was not due to a decrease in fecundity (egg laying). After 6–8 weeks, TM3, *ry* ^RK^ *Sb* ^1^ *Ser* ^1^/TM6B, *Tb* ^1^ flies laid large numbers of eggs upon returning to 25°C, but only a small fraction of the embryos developed. To test for sex-specific limits on fertility, the remaining females (2–5/vial) and males (3–5/vial) were separated at the end of the third brood and crossed to young (1–2 d) male or virgin female *y w* flies for 1 additional brood of 6 d duration. Rather than a sex-specific effect, the old females and old males from some individual vials were able to generate much larger numbers of progeny when crossed with young flies than during the preceding broods with each other, whereas 1 or both sexes in other vials had become completely sterile. The same procedure was performed with the 2S12i7Y *w* flies after 6–8 weeks, with similar results. These results suggested that the generation time might also be extended by cooling in less fertile genotypes, but only for brief intervals under conditions where further curtailment of reproduction could be tolerated.

**Table 15:** Fertility (TM3, *ry* ^RK^ *Sb* ^1^ *Ser* ^1^/TM6B, *Tb* ^1^) After Housing Flies at 11°C (0–12 Weeks).

Source of Variation	Type III SS*	df**	Mean Squares	F ratio	P value
Time (0–12 Weeks)	32 642.833	6	5 440.472	134.820	<0.0005
Brood (1–3)	689.060	2	344.530	8.538	0.001
Time×Brood	1 093.953	12	91.163	2.259	0.019
Error	2 501.917	62	40.353		

*SS = sum of squares. **df = degrees of freedom.

## Discussion

The broad objective of this study was to explore methods by which the amount of time invested in *Drosophila* stock maintenance could be reduced. In the *y w* lineage, reproduction could be postponed at least 20 d by storing adult flies at 8°C or up to 12 weeks at 11°C. Parental fertility during the first 2 d of the recovery phase at 25°C was very sensitive to cooling and overall fertility decreased progressively as the time of storage increased, but the fertility of F1 progeny was not compromised. In the commonly used laboratory strain, *w* ^1118^, the generation time was extended up to 60 d by storage at 11°C; however, TM3, *ry* ^RK^ *Sb* ^1^ *Ser* ^1^/TM6B, *Tb* ^1^ flies were resistant to housing at 11°C for no more than 4 weeks.

An initial experiment was conducted at 4°C to identify developmental stages that were most resistant to chilling. Consistent with past studies [10], [20], adults and pupae were clearly hardier than embryos and larvae. The death and necrosis of larvae, particularly in the third instar, suggested strongly that a healthy stock could best be maintained by separating pupae or adults from larvae during extended intervals of cold storage. The adults lay on the food surface during chill coma (4°C), and in subsequent experiments they stood on the food and sides of the vials during the quiescence induced by milder cooling (8–11°C). Observations of their recovery at 25°C and of flies that eclosed after chilling at 4°C as pupae suggested that survivorship would be promoted by removing the flies from churned food and placing them in clean vials with fresh food prior to chilling. Given the absence of any difference in survivorship between pupae and young adults, and the greater ease of removal of adults from vials used for development, only adults were studied subsequently.

The selection of an appropriate temperature range is vital to the success of long-term cold storage. Both the longevity and fertility of flies are strongly temperature-dependent, with maxima at roughly 10°C and 25°C, respectively [21]–[23]. Some researchers already maintain fly stocks continuously at temperatures as low as 18°C to prolong the generation time [24]; however, certain phenotypes are affected by the developmental temperature [25], [26] and egg-to-adult viability is diminished [27]. To postpone rather than slowing reproduction, and avoid mixing developmental stages, the temperature would need to be lowered to no more than ∼12°C [17], [24]. At the lower end of the range, mortality in this study reached 100% within 10 d at 4°C, but it was minimal for up to 20 d at 8°C or 12 weeks at 11°C. Reproductive output was nil at 11°C, but flies retained the ability to reproduce at 25°C following incubation at 4–11°C. Consistent with past studies [16], [28], prolonged incubation at low temperatures diminished the number of F1 progeny. The decrease was much faster as a function of storage time at 4°C than 8°C, and appreciably faster at 8°C *vs*. 11°C. Narrower temperature increments from 9–12°C could be compared in a future study, to establish a more exact maximum and tolerance range.

Adult fertility was measured using methods intended to mimic conditions of regular stock maintenance, minimize the amount of effort that would be required, and assess the likelihood of sustaining viable stocks in the laboratory as realistically as possible. In particular, fully eclosed, adult progeny were counted, rather than embryos or larvae, because only flies that completed development could contribute to subsequent generations. In the case of TM3, *ry* ^RK^ *Sb* ^1^ *Ser* ^1^/TM6B, *Tb* ^1^ flies, a count of embryos would have overestimated the viability of the stocks after storage at 11°C. Use of F1 survival to adulthood as a minimum criterion of stock viability led to a more cautious conclusion that prolonging the generation time might jeopardize the survival of sensitive stocks. Also, fertility was tested using the regular vials and food medium on which stocks are normally maintained, rather than agar plates. Counting adults made this method feasible, whereas larvae would have been difficult to count after they began burrowing in the opaque medium. The use of carbon dioxide in the collection of parental flies is most unlikely to have affected either their resistance to cooling or subsequent fertility, given that (i) the very young adults that are most sensitive to this form of anesthesia [24] were not collected and (ii) the duration of exposure was extremely brief (≤5 min) and recovery times before cooling were very long (typically ∼24 h), in comparison with experiments in which CO_2_ was shown to have only transient effects on cold tolerance [29] and no effect on fertility [28].

In most experiments, *y w* and *w* ^1118^ fertility was recorded for the first 6 d of the recovery phase at 25°C, which was long enough to detect any transient fluctuations in the immediate aftermath of cooling. Division of this 6 d interval among multiple broods revealed that the decline in fertility induced by storage at 4–11°C would have been overestimated substantially by recording for only 1–2 d after the flies returned to 25°C. Similarly, rapid cold hardening was followed in the short term (8 h) by a large decrease in reproductive output (F1 embryos that developed to adulthood) [30], but fecundity over a longer recovery interval (5 d) was unaffected [31]. Dead parental flies were not replaced between broods, because the key issue was their ability to generate progeny, which would be affected equally by death or infertility. The 6 d interval was also short enough to leave some reserve capacity for reproduction, thereby yielding conservative estimates of the duration of cold storage consistent with stock preservation. Indeed, additional reproductive output after >6 d recovery permitted the *w* ^1118^ population to reach the replacement threshold after 60 d at 11°C, and the weaker TM3, *ry* ^RK^ *Sb* ^1^ *Ser* ^1^/TM6B, *Tb* ^1^ and 2S12i7Y *w* stocks to do so for up to 4 and 10 weeks, respectively, but it would be prudent not to extend cold storage to these limits. Lastly, in the second experiment with *y w* flies, a single brood of 6 d duration yielded as many progeny as 3 broods of 2 d duration after storage at 11°C for 8 weeks (but not 0 weeks, presumably due to exhaustion of resources). This result indicates that the time saved by storing flies at 11°C would not have to be repaid by performing multiple broods after the flies were returned to 25°C. Unexpectedly, the results showed that even provision of a new vial after storage could be omitted without compromising either the number or fertility of F1 flies on standard medium.

Parallel groups of unmated *y w* flies were held at 11°C and 25°C, in order to compare the rates at which cooling and aging affected their fertility. Short-term cooling (1–4 weeks) had a more severe impact than aging, but only during the first 2 d of the recovery phase. After longer-term storage (5–8 weeks) and during days 2–6 of recovery, the decrease in reproductive output due to cooling was never greater and frequently less than the attrition due to aging at 25°C. Flies housed at 11°C for 10 weeks generated significant numbers of progeny upon their return to 25°C, whereas flies housed at 25°C for 10 weeks before crossing were completely sterile. After 12 weeks, which is longer than the mean life span of this strain at 25°C, the flies housed at 11°C still retained sufficient reproductive capability on average (≥1 adult F1 fly per parent) to sustain the stock. Thus, cooling caused a sharp initial decline in fertility, but subsequently the rate of decline was slower, presumably due to a slower rate of aging at 11°C. It should be noted that separating flies by sex just after eclosion and holding them at 25°C was not intended as a practical way to make stock maintenance take less work.

Food spoilage was an unexpectedly minor issue throughout the study. *y w* flies were housed at 8°C with or without food, to test the hypothesis that the absence of food would constitute a milder stress than food spoilage during prolonged cold storage. The absence of food during the first 2 weeks at 8°C had no effect on subsequent reproductive output at 25°C. After longer intervals, the flies with food retained the ability to recover and reproduce, whereas the flies in empty vials died of starvation and/or desiccation. At 11°C, mold spots appeared on the food surface after 6–12 weeks, but they did not affect subsequent fertility at 25°C. Fertility was also equivalent whether 3 males and 3 females were housed separately or together at 11°C, indicating that doubling the number of flies from 3 to 6 did not lead to more rapid food spoilage, and that separating the flies by sex prior to cold storage was not necessary. It remains to be determined whether a greater population density during storage would accelerate deterioration of the food. Additionally, the standard amount of mold inhibitor (MP) was enough to preserve the food for as long as the flies could sustain some reproductive capacity at 11°C. Increasing the concentration of MP, ethanol diluent or both had neutral or negative effects on the yield of F1 progeny after 0 weeks at 11°C, as well as survivorship of parents and yield of both F1 and F2 progeny after incubation for 8 weeks at 11°C.

Development of F1 progeny to adulthood provides some evidence that a stock could be stored temporarily at a cool temperature, but it might be hypothesized that the fitness of subsequent generations would be compromised. With respect to reproductive capability, this hypothesis was tested by performing crosses among virgin F1 progeny of *y w* parents housed at 11°C for 8 or 10 weeks. The yield of adult F2 progeny was equal to or greater than the yield from F1 progeny of parents aged at 25°C, and the yields of F1 adults from young parents and from young *y w* flies obtained from stock bottles on a standard, 2-week generation cycle. Thus, prolonged housing at 11°C for one generation had no obvious effect on longer-term fitness.

Several additional questions must be addressed before cold storage of fly stocks can become routine. First, would repeated exposure to a cold environment for multiple generations lead to a weakening of survivorship or fertility that is not apparent after only a single generation? Second, for any laboratory affected by fly mites, would prolonged storage of flies and media at 11°C favor the mites? Third, would long-term changes in conditions of stock maintenance affect gene expression and associated phenotypes? Long-term (20 years) housing at varying temperatures had effects on heat-shock transcription factor expression that persisted after acclimation at 25°C [32]. Indeed, even short-term cooling (1 generation of development at 15°C or 5 d cooling of young adults at 11°C) had drastic and contrasting effects on resistance to cold and warm field conditions [33]. Other phenotypic effects that might be predicted include changes in the abundance of numerous metabolites, as occurs with larger temperature drops during rapid cold hardening or cold shock [30]. Fourth, would the cold environment impose selective pressure at loci affecting traits other than survival and fertility? Such selection might be expected, given the marked variation in resistance to cold stress indicated by varying chill coma recovery times among genotypes of *D. melanogaster* [34]. The decrease in fertility after prolonged incubation at 11°C implies that some progeny that would have contributed to subsequent generations without the delay were not produced. If allele frequencies differed between those progeny that ‘survived’ and those that did not, then artificial selection for cold hardiness occurred. Such selection might lead to more rapid genotypic or phenotypic change than random mutation accumulation and genetic drift on a 2-week generation interval at 25°C. The rate of selection would be predicted to vary among stocks, being greatest for outbred and least for highly inbred stocks, in which new mutations would be the only source of allelic variation. The importance of artificial selection would also vary according to the purpose for which stocks were kept. For instance, selection would be of greater concern in stocks used for behavioral studies than stocks in which single-gene mutations were maintained over balancer chromosomes for outcrossing to other backgrounds. Conversely, maintaining flies at low temperatures has been shown to decrease the somatic mutation rate [35]. If the rate of heritable mutation accumulation is proportional to the number of generations per year [4] rather than the passage of chronological time, then the use of cold storage to prolong each generation should allow genotypes, if not phenotypes to be retained for longer than continuous maintenance at 25°C.

Lastly, what physiological changes underlie the decrease in fertility during cold storage? Depending on the answer, perhaps the interval between generations can be extended even longer. Fertility is affected strongly by the abundance of particular nutrients, especially methionine [36]. The relatively nutrient-lean medium used in this study might be ideal for cold storage, but either supplementation during the cooling phase or transfer to a more nutrient-rich medium upon return to 25°C might promote greater reproductive output. The ideal composition of the medium is not intuitively obvious, given that the effects of nutrients appear to be sex-specific and inconsistent among studies [37].

Allowing a slightly cautious 50 d for cold storage, 6 d for egg laying and 10–14 d for development, the interval between generations of *D. melanogaster* stocks could be extended to 70 d using methods accessible to investigators for whom cryopreservation of embryos is not feasible. At present, drawbacks would include a diminished number of F1 progeny and potential for alteration of genotypes through artificial selection for cold hardiness. The latter subject should be explored before cold storage can be recommended for routine maintenance of stocks. Nevertheless, the results of this initial study are sufficiently promising to warrant investigation of these issues.
